# The role of animal hosts in shaping gut microbiome variation

**DOI:** 10.1098/rstb.2023.0071

**Published:** 2024-05-06

**Authors:** Elisa Maritan, Andrea Quagliariello, Enric Frago, Tomaso Patarnello, Maria Elena Martino

**Affiliations:** ^1^ Department of Comparative Biomedicine and Food Science, University of Padova, 35020 Padova, Italy; ^2^ CIRAD, UMR CBGP, INRAE, Institut Agro, IRD, Université Montpellier, 34398 Montpellier, France

**Keywords:** host–microbe interaction, gut microbiota, holobiont

## Abstract

Millions of years of co-evolution between animals and their associated microbial communities have shaped and diversified the nature of their relationship. Studies continue to reveal new layers of complexity in host–microbe interactions, the fate of which depends on a variety of different factors, ranging from neutral processes and environmental factors to local dynamics. Research is increasingly integrating ecosystem-based approaches, metagenomics and mathematical modelling to disentangle the individual contribution of ecological factors to microbiome evolution. Within this framework, host factors are known to be among the dominant drivers of microbiome composition in different animal species. However, the extent to which they shape microbiome assembly and evolution remains unclear. In this review, we summarize our understanding of how host factors drive microbial communities and how these dynamics are conserved and vary across taxa. We conclude by outlining key avenues for research and highlight the need for implementation of and key modifications to existing theory to fully capture the dynamics of host-associated microbiomes.

This article is part of the theme issue ‘Sculpting the microbiome: how host factors determine and respond to microbial colonization’.

## 1. Introduction

All animals are chimeric creatures, covered inside and out with microorganisms, collectively called ‘microbiota’. The collection of genomes of such microbial communities, together with their structural elements and metabolites, is referred to as the ‘microbiome’ [1]. Host-associated microbiomes make essential contributions to animal health by extracting nutrients from dietary substrates, promoting host development, stimulating the immune system, and protecting the host from invasion by pathogens and other natural enemies. In return, the host provides a nutrient-rich environment and additional defence systems against microbial competitors [2].

One of the central goals of host–microbe research is to understand the ecological factors that drive the composition of host-associated microbiomes. Traditionally, researchers have used theoretical models and experimental observations to ask under what conditions *local* dynamics (selection by host factors) outweigh *neutral* dynamics (i.e. transmission, drift, priority effects) in explaining variation in host microbiomes. In this regard, host factors, such as developmental stage and genetic background, have been shown to influence gut microbiome composition across different animal species (e.g. humans, mice, zebrafish, chicken, cattle, swine) [3]. At the same time, neutral models and environmental factors (e.g. diet, biogeography, drugs) have been found to best describe gut microbiome composition in animals, including humans [4]. However, despite large-scale sampling efforts, the relative contribution of such ecological factors and how they converge to determine microbiome variation across animals is still elusive. As a result, the main forces that determine host-associated microbiome variation remain poorly understood.

In this review, we summarize our current understanding of how host-related factors drive variation in the gut microbiome. Our goal throughout is to highlight conserved mechanisms as well as differences in the complex host-level selection of microbiota composition across animal hosts. To break down this complexity, we focus on four main host-related factors: anatomy, genotype, vertical transmission and immune response. Finally, we discuss the direction of future research in host–microbe symbiosis and the importance of integrating concepts and approaches to explain the dynamics of host–microbe interactions.

## 2. Anatomy

**Figure 1.**
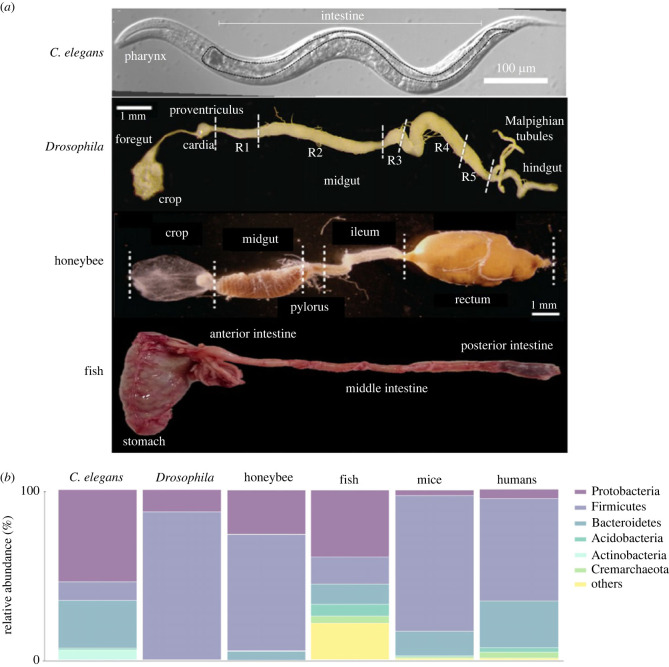
(*a*) Differential interference contrast microscopy (DIC) image of the body of *C. elegans* [5] and dissected guts of adult *Drosophila* [6], honeybee [7] and *Seriola dumerilii* [8]. The different gut regions are labelled in each figure. (*b*) Relative abundance of the major gut microbiota phyla across invertebrates (i.e. *C. elegans* [9], *Drosophila* [10], honeybee [11]) and vertebrates (i.e. fish [12], mice and humans [13]).

Microbiome assembly in animal hosts begins at birth and, during host development, it undergoes a variety of dynamic processes that influence its establishment, function and evolution. One of the main factors explaining the variation in gut microbiome across animals is based on their anatomical differences. Organ and epithelial peculiarities (e.g. length, surface area, transit time), together with variations in physico-chemical conditions (e.g. pH, redox potential, oxygen availability, antimicrobial compounds) along the gastrointestinal (GI) tract are crucial regulators of microbial homeostasis, shaping the composition, density and colonization rate of gut microbiome across vertebrate and invertebrate taxa (figure 1*a* and table 1).

**Table 1.** Main anatomical factors shaping gut microbiome composition across the main hosts covered in the review. *C. elegans*, *Caenorhabditis elegans*; P, present; A, absent; –, not available; TLR, Toll-like receptor; NLRs, NOD-like receptors; AM, antimicrobial; AMPs, antimicrobial peptides; fip, fungal-induced peptides; DUOX, dual oxidase; PGRPs, peptidoglycan recognition proteins; MAMPs, microbe-associated molecular patterns.

host	anatomical features			physico-chemical/molecular factors
	epithelial structure	niche	metamorphosis	
*Hydra*	glycocalix with mucus-like properties [14]	A	A	antimicrobial peptides (hydramacin, periculin, arminin peptide families); TLR-domain-containing protein precursors NLRs [15]
*C. elegans*	peritrophic matrix [16]	P [17]	A	AM effectors (lysozymes, caenacins/neuropeptide-like proteins, C-type lectin domain-containing proteins, defensin-like AMPs, fip and fip-related peptides, thaumatin-like proteins [18]
				DUOX system [19]
				Tol-1, pathogen avoidance behaviour [20]
*Drosophila*	peritrophic matrix [21]	P [22]	P [23]	AMPs (drosocin, diptericin and drosomycin) [24]
				PGRPs [25]
				DUOX system [26]
				TLRs, not directly involved in MAMPs recognition [27]
honeybee	peritrophic matrix [28]	P [29]	P [23]	AMPs (apidaecin Ia, apidaecin Ib, apidacein 2, apidacin, abaecin, defensin-1, defensin-2, hymenoptaecin, jellein 1, jellein 2, jellein 4)
				PGRPs, TLRs [30]
fish	mucus layer [31]	–	A	AMPs [32,33]
				DUOX system [34]
				NLRs, TLRs [35]
				bile acids [36]
				B cells [37]
				T cells [38]
mouse	mucus layer [39]	P [40]	A	PGRPs [41]
				AMPs [42]
				DUOX system [43]
				NLRs [44]
				TLRs [45]
				bile acids [46]
				B cells [47]
				T cells [48]
human	mucus layer [49]	P [50]	A	PGRPs [51]
				AMPs [52]
				DUOX system [53]
				NLRs [44]
				TLRs [45]
				bile acids [54]
				B cells [55]
				T cells [56]

All animals, including humans, share significant microbiome heterogeneity along the intestine, with microbes distributed along a gradient of intensity, starting from the low numbers of microbial cells per gram of gut content in the upper GI regions to significantly higher values in the distal parts of the gut. In the upper GI regions, this distribution is mainly driven by the acidic conditions (luminal pH < 3), coupled with the high concentrations of host secretions (e.g. antimicrobial effectors, bile acids, pancreatic fluids) and short transit times (tables 1 and 2) [69]. In insects, pH may be an important determinant of differences in gut microbial communities among insect groups. Caterpillars of butterflies and moths, for instance, have guts that are very poor in terms of microbial diversity, some studies suggesting that this group does not have resident microbes. One of the reasons is thought to be the harsh conditions of fast food passage and high pH levels in the midgut [70]. Studies in *Drosophila melanogaster* (hereafter referred to as *Drosophila*) have shown that genetic ablation of the copper cells (i.e. the cells of the acidic compartment of the midgut) or of the V-ATPase, which mediates the acidification of this region, results in increased pH and a higher abundance of gut microbes in both larvae and adults [59,71]. Acidification of the surrounding environment is also used by commensal bacteria as a mechanism to control microbial invasion and protect the host [72]. On the contrary, the increase in pH, the high transit time in the ileum and rectum and the resulting accumulation of food content favour microbial proliferation [29] (table 2). Similar to the microbial heterogeneity of the GI axis in insects and mammals, a general progressive increase in the bacterial population size and variation in community composition from the stomach to the hindgut has also been observed in fish [73] (table 2). However, in contrast to mammals or insects, where microbial diversity is highest in the hindgut, in fish, the highest overall diversity is found in the midgut [74]. This result may be due to differences in the dominant microbial taxa between fish and other vertebrates (i.e. reptiles, birds and mammals): while the latter are mainly dominated by Bacteroidetes and Firmicutes, the microbiome in fish is mainly composed of Proteobacteria and Firmicutes [12] (figure 1*b*). It has been shown that selective pressures arising from a wide range of host factors, including host anatomical features, play a unique role in the ecology of fish microbiomes [74]. Starting with the seminal study by Roeselers *et al*. showing striking similarities between the gut microbial composition of laboratory-reared and wild-caught zebrafish [75], host-mediated selection of gut microbiome has been observed in many fish species, including European seabass (*Dicentrarchus labrax*), Atlantic cod (*Gadus morhua*), rainbow trout, salmon and several carp species (i.e. grass carp, crucian carp and bighead carp) [76,77]. However, the specific mechanisms responsible for such selection lag behind our knowledge of those operating in humans and other mammals, largely owing to the high biodiversity of fish and, consequently, the high variation in morphology and function of their GI tract [73,74,78].

**Table 2.** Physico-chemical and molecular factors shaping the gut microbiome composition across the main hosts covered in the review*.* For each gut section, pH, oxygen and microbial load are reported. CFU, colony-forming units; *C. elegans*, *Caenorhabditis elegans*; –, not available.

	gut section					
*pharynx*			*intestine*			
host	pH	CFU	pH	CFU		
*C. elegans*	5.96 ± 0.31 [57]	–	3.59 ± 0.09 [57]	*ca* 10^2^ worm^−1^ [58]		
	*foregut*		*midgut*		*hindgut*	
	pH	CFU	pH	CFU	pH	CFU
*Drosophila*	*ca* 7 [59]	10^4^ mm^−3^ [22]	midgut: *ca* 7–9; copper cell region: <3 [59]	10^3^ mm^−3^ [22]	*ca* 5 [59]	10^3^ mm^−3^ [22]
honeybee	4.82 ± 0.08 [60]	10 (total copies of 16S rRNA) [29]^3^	5.6–7 [60]	10^4^ (total copies of 16S rRNA) [29]	ileum: 5.1–6.7; rectum: 5.2–5.3 [61]	10^8^–10^9^ (total copies of 16S rRNA) [29]
	*stomach*		*small intestine*		*large intestine*	
	pH	CFU	pH	CFU	pH	CFU
fish	4.2–5.2 [62]	*ca* 2.2 × 10^3^ g^−1^ [63]	7.6–8.6 [62]	3.1 × 10^3^ g^−1^ [63]	8.2–8.7 [62]	10^4^ g^−1^ [64]
mouse	2.7–4.1 [65]	*ca* 10^3^ g^−1^ [66]	*ca* 5 [65]	10^4^–10^7^ ml^−1^ [66]	7.0–7.6 [65]	10^9^–10^10^ ml^−1^ [67]
human	1.0–2.5 [68]	*ca* 10^3^–10^5^ g^−1^ [69]	6.0–7.4 [68]	10^8–^10^9^ g^−1^ [69]	colon: 7.1–7.5; caecum: 5.6; rectum: 7.4 [68]	colon: 10^8^–10^11^ g^−1^ [69]

Another critical factor shaping the composition and variation of the gut microbiome in the GI tract is the redox potential of the gut lumen. In contrast to the extensive anoxic regions of the mammalian gut, the *Drosophila* gut epithelium is endowed with an aerobic metabolism owing to an extensive network of tracheal cells that allow oxygen transfer [79]. Here, oxygen diffusion from the epithelium results in a predominantly oxic/hypoxic gut lumen, which is favourable for aerobic/aerotolerant microorganisms, but hostile to obligate anaerobes [79]. In addition, dietary microbes (e.g. *Lactobacillus* spp.) are able to induce the NADPH oxidase DUOX production of epithelial reactive oxygen species (ROS), which in turn are involved in controlling their density in the gut [80]. In mammals, variations in redox potential along the GI tract directly control microbial composition and metabolic capacity. Whereas in the absence of oxygen, obligate anaerobic bacteria catabolize complex carbohydrates into fermentation products (e.g. short-chain fatty acids) that contribute to host nutrition, in the presence of oxygen, facultative anaerobic bacteria catabolize fermentation products into carbon dioxide, which would instead interfere with host nutrition. Thus, to ensure that the microbiome remains beneficial, the host maintains colonocytes in a state of hypoxia, ensuring the dominance of obligate anaerobic bacteria [81].

Beyond physico-chemical factors, the animal intestinal epithelium provides a physical barrier that contributes to maintaining a balance between protecting the host from pathogens and tolerating beneficial microbes. In the tubular body structure of *Hydra*, microorganisms must overcome the physico-chemical barrier represented by the multilayered glycocalyx covering the ectodermal epithelium [14]. The glycocalyx, which is also conserved in the nematode *Caenorhabditis elegans* [16], has two functionally distinct compartments: an inner stratified layer that acts as a physico-chemical barrier and produces vast amounts of antimicrobial peptides (AMPs) (table 2), and an outer layer composed of constantly renewed transmembrane glycoproteins, proteoglycans and glycolipids, which provide the habitat for the symbiotic bacterial community [82]. For this reason, it has been proposed that the outer layer of *Hydra*'s glycocalyx (as potentially in other animals) has mucus-like properties rather than being a part of the membrane-anchored glycocalyx. This anatomical organization likely functions as a defence because bacteria have never been observed to reach the dense inner layers of the glycocalyx or even the ectodermal cell membrane [14]. Strikingly, a similar observation was made in the mammalian colon. An inner firmly adherent layer with a stratified organization was devoid of bacteria, whereas the outer loose layer appeared to be colonized by symbionts [83]. In insects, an anatomical barrier is provided by the very close apposition between the epithelial cells, mediated by septate junctions, which are functionally equivalent to the tight junctions in the mammalian gut epithelium [79]. In addition, insects appear to have evolved specific epithelium-associated mechanisms that effectively separate the microbes from the host tissue, presumably as adaptive strategies to further ensure microbiome control in the absence of the adaptive immune system of higher metazoans. The most evident example of such adaptations in most insects is provided by the peritrophic membrane, a tightly arranged semi-permeable membrane that protects the foregut and hindgut and prevents the translocation of pathogenic microorganisms and microbial toxins to the epithelium [84]. The peritrophic membrane consists of chitin-binding proteins that are extensively glycosylated and structurally similar to the mucins of the vertebrate mucus. It has been suggested that the *Drosophila* microbiome, like some mucus-associated bacteria in the mammalian gut, may directly interact with these proteins [85]. In the distal GI tract (e.g. the proximal colon in mammals), the mucosal biofilm formation is indeed conserved from mammals to amphibians, albeit with structural differences, suggesting an ancient evolutionarily conserved origin of this region as a barrier and habitat for the microbiome [14]. A divergence in microbial composition between the mucosal and digesta-associated colonic communities has been observed in several mammals, including humans [86], macaques [87], mice [88], cows [89] and flying squirrels [67]. However, some bacteria can penetrate the mucus and bind directly to the epithelium. *Acinetobacter* and Proteobacteria have been found in a significant proportion of the small intestine and colonic crypts in healthy mice [40] and humans [90]. Some invertebrates are also endowed with specific anatomical niches that favour the colonization of beneficial microorganisms and allow them to exert strong control over their associated microbes through compartmentalization [91–93]. Many examples come from insects, a well-studied one being the bean bug, *Riptortus pedestris*, which orally acquires a specific *Burkholderia* symbiont, which forms dense colonies in midgut crypts [94]. A similar physical niche is also created in the adult *Drosophila* foregut, which is specifically colonized by wild beneficial strains of *Lactobacillus* and *Acetobacter* strains. Bacterial colonization appears to be favoured by the fly itself in a highly specific manner, involving specific molecules (probably mucins) in the extracellular matrix of the proventriculus that are able to bind exclusively to the bacterial surface of colonizing competent strains, but not to non-colonizing strains [22]. In the fall armyworm (*Spodoptera frugiperda*), the protective layers of gut microbes can be weakened by toxic plant chemicals, thus altering the protective peritrophic matrix and ultimately allowing gut residents to leak into the body cavity and cause disease [95].

Age and stage of development are other common factors that contribute to microbiome variation in different animal hosts. This has largely been demonstrated in honeybees [96], *Drosophila* [97], fish (both wild and aquaculture) [98], mice and humans [99]. Particularly in humans, the diversity of the microbiome increases with age and becomes fixed at around 3 years of age, when the composition of the gut microbiome more closely resembles that of adults [99]. Notably, in many holometabolous insects (i.e. beetles, flies, wasps, ants, bees, butterflies, moths and others), metamorphosis also imposes several constraints on the assembly and persistence of gut microbes [23]. In many insects, the larval gut (including the microbes in the gut lumen) is purged prior to pupation and newly emerging adults excrete the remnants of the larval gut as meconium [100]. This perturbation can cause gut-associated symbionts to be relocated within the host, suppressed or lost altogether [101]. Similar restructuring of the microbiome occurs in other animals undergoing metamorphosis, such as lampreys [102], frogs [103] and sponges [104].

Finally, studies in both invertebrate and mammalian model systems have demonstrated sex-specific differences in the composition of the gut microbiome [105,106]. In mammals, this has been linked to differences in sex steroid hormones between males and females [107]. However, the noise introduced by confounding factors such as diet, age and host genetic background has obscured sex differences in many different model systems [105].

**Figure 2.**
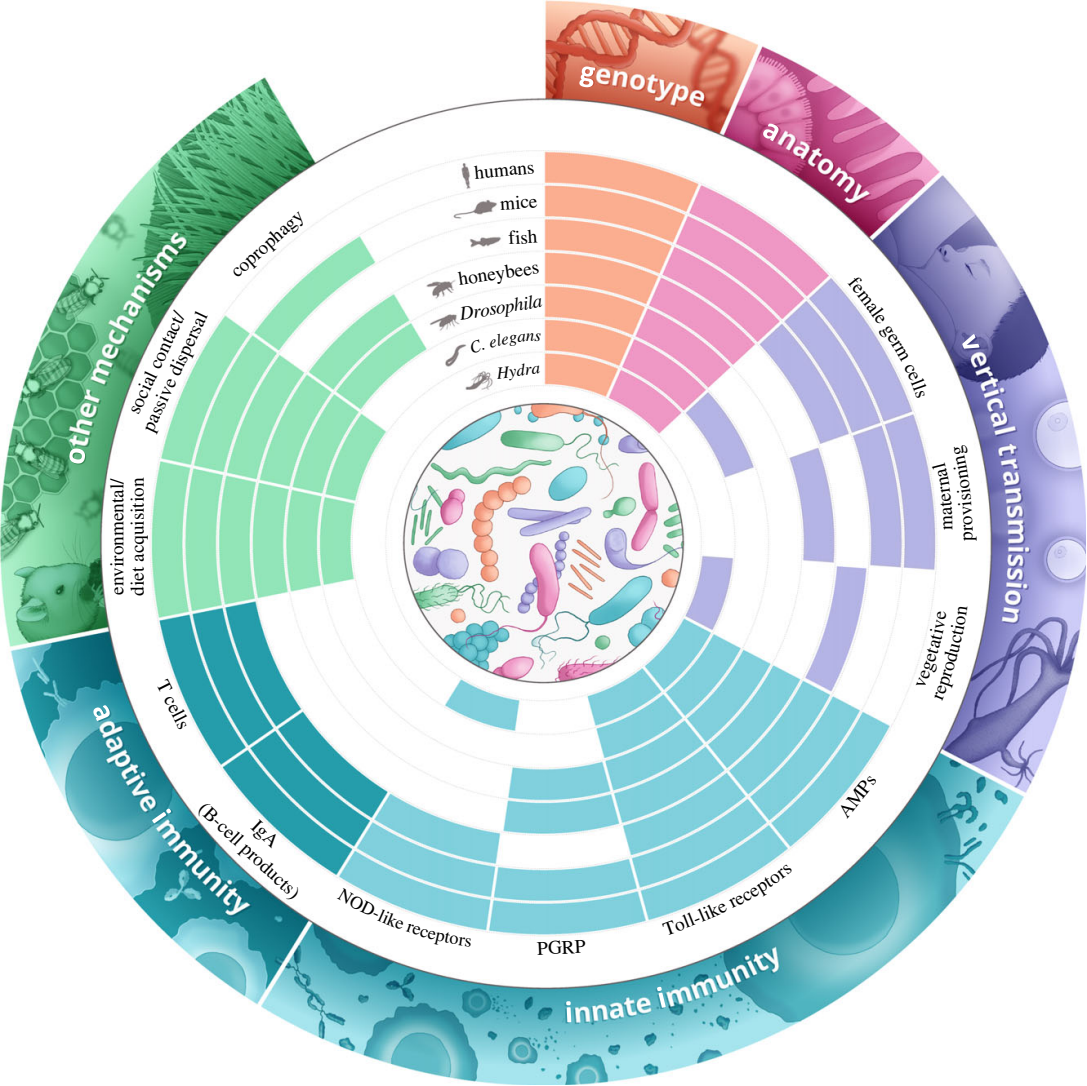
Summary of the main host-related (genotype, anatomy, vertical transmission, immunity) and environmental factors driving gut microbiota variation across animals. Each circle refers to the main host species covered in the review. Coloured cells indicate the presence of the respective factors, while empty cells indicate the absence of the factor in the respective host. Further details on each factor can be found in tables 1 and 2. *C. elegans*, *Caenorhabditis elegans*; AMPs, antimicrobial peptides; PGRP, peptidoglycan recognition protein; IgA, immunoglobulin A. Image credits: Joana C. Carvalho.

In summary, although animals carry different microbial species in their gut, anatomical features (e.g. pH gradient along the gut, redox potential, developmental stages) represent conserved factors that shape the ecology of the gut microbiota in all animals (figure 2).

## 3. Genotype

Much of the research on host–microbe symbioses has focused on understanding the impact of host genetics on the composition of animal-associated microbiomes. To this end, research studies have primarily used genome-wide association studies (GWASs), quantitative trait locus (QTL) analyses and 16S rRNA gene amplicon sequencing. Host genotype has been found to be significantly associated with gut microbial composition in a wide range of species and taxa, from nematodes to mammals. Under environmentally controlled conditions, QTL mapping studies coupled with 16S rRNA gene sequencing and the use of inbred mouse strains and reference populations showed that host genetic variation can explain a substantial proportion of the variation in gut microbiome composition (i.e. from 1.6 to 9%). This suggests a symbiotic relationship that has co-evolved over millions of years whereby hosts filter required microbes [108]. Such an estimate appears to be conserved in humans, where host genetics has been estimated to explain between 1.9 and 8.1% of variation in the gut microbiome [4,109]. By performing 16S gene sequencing and functional assessment of the gut microbiome in different *Caenorhabditis* species, spanning a time frame of 200–300 Myr of evolution, Berg *et al*. [110] observed a consistent clustering of microbiome based on genotype. In addition, a study by Zhang *et al*. exposed genetically distinct *C. elegans* strains to a 63-member model microbiome and showed that several genomic regions are associated with the abundance of specific microbial taxa across host strains, with the most significant overlap observed for genes involved in host insulin signalling pathways [111].

In *Drosophila*, research studies investigating the link between specific genes and the microbiome have identified several immune-related genes (e.g. *nubbin*, *Drosophila* homologue of the mammalian transcription factor Oct1/Pou2fl, *caudal*), as well as genes involved in neural and cellular growth and development, as key regulators of gut microbial colonization [112,113]. At the same time, by using 36 fly lines from the Drosophila Genetic Reference Panel (DGRP), Early *et al*. showed that gut microbiome variation in *Drosophila* commensal bacterial load, both in composition and abundance, can be largely attributed to physical aspects of gut cell growth and development, including neuronal function, neuronal morphogenesis and development [114]. A GWAS conducted crossing different bacteria species and strains and honeybee genotypes revealed a significant association between gut microbiome composition, particularly *Bifidobacterium* spp., and host receptor genes, such as the glutamate receptor gene and the G protein-coupled receptor gene, specifically expressed in the bee brain [115]. While these studies are primarily based on associations between host genes and the presence of specific gut bacterial species, experimental work studying host control over gut microbial structure are also popular. Reciprocal transplantation of gut microbiome into germ-free (GF) zebrafish and mouse recipients has shown that the transplanted communities retain similarities to the original community in terms of the lineages present. However, the relative abundance of these lineages changes to resemble the normal gut microbial community composition of the recipient host [116]. Other studies have shown strong correlations between fish host genotype and microbiome composition in stickleback populations (where more genetically diverse populations have more diverse gut microbiomes) [117], cyprinids [118] and salmonids [119], and on hybrid individuals [120]. Specifically, by using 16S rRNA gene sequencing on fish lineages derived from parents with different feeding habits (i.e. the herbivorous *Megalobrama amblycephala*, and the carnivorous *Culter alburnus*), Li *et al*. discovered a strong correlation between genotype and gut microbial assemblages. The dominant microbial taxa showed a significantly positive correlation with the genetic factors of both reciprocal hybrids and both parents. These findings not only suggest that host genetics significantly influence gut microbial communities, but also imply that genomic interactions may directly or indirectly influence the dietary adaptation and evolution of fish, and ultimately shape the composition of the gut microbiome [120].

Heritability studies have been widely used to estimate the extent to which host genetics contribute to variation in the gut microbiome. In humans, the heritability of gut microbiome was initially demonstrated in twin studies. These studies assumed that if the genetic background of the host influences a particular phenotype, measures of the phenotypic trait of interest would be more similar within monozygotic twin pairs than within dizygotic twin pairs [121]. However, the research carried out on twins has so far been inconclusive, with several studies coming to different conclusions [122]. Among the different microbes studied, some have stronger heritabilities, for example those in the family Christensenellaceae (phylum Firmicutes) [123]. In addition, GWAS approaches, and more recently ancient DNA data (box 1), have been used in large populations of unrelated individuals worldwide to explore associations between host genetic variants, gut microbial species, and phenotypic traits. Examples of human genes found to be associated with gut microbiome composition include the vitamin D receptor [129], the *ORA6A2* gene responsible for the soapy taste of coriander experienced by some individuals, *CD36* associated with the ability to taste long-chain fatty acids on the tongue [109], and immune genes, such as *ABO* and *FUT2* [130,131]. However, the most consistently replicated association to date is between genetic variants of the lactase gene (*LCT*) and the abundance of *Bifidobacteria* in the human gut [3,132]. (For extensive details about this topic, refer to [133].) Interestingly, this association is specifically observed in individuals who report consuming dairy products, highlighting a gene-by-environment interaction involving the microbiome.

Box 1. **The contribution of ancient DNA to understanding host-mediated selection on microbiome evolution.**

Over the past three decades, the field of ancient DNA (aDNA), recently honoured by the Nobel Prize to Svante Pääbo, has dramatically improved our technical ability to reconstruct our past. It is now possible to investigate specific key steps in the evolution of different organisms, including host microbiomes, and to obtain direct information on the co-evolutionary relationship between host and microbes [124]. Using ancient coprolite data, a recent study by Sanders *et al*. has shown how humans, along with other non-human primates, co-evolved with their symbionts [125]. The collected evidence shows that the gut microbial community in humans has experienced strong selective pressures linked to genetic and physiological changes that have occurred during our evolution as a species, but which are independent of the population's lifestyle. In total, 10 different bacterial phyla were found to have co-diversified during our evolution, with varying degrees of covariation between phyla. Human-associated microbes showed signals of strong positive and purifying selection with respect to pan-associated symbionts, with several clusters of orthologous genes (COGs) showing significant co-diversification signatures. This supports the hypothesis that, despite bacterial phylogenetic history, multiple bacterial functions have been selected for over millions of years. The human oral microbiome also appears to show signals of co-evolution with the host. Evidence from aDNA studies has highlighted the existence of 10 different bacterial genera that have been maintained throughout the evolutionary history of all African hominids, suggesting that they are likely to have been important members of the oral environment since around 40 Ma [126]. In addition, as reported for the gut microbiome, the *Homo* lineage (including Neanderthals) shows functional and taxonomic differences from non-human primates. An impressive example of the co-evolution of *Homo* microbes came from the analysis of ancient and modern oral streptococci. Indeed, human *Streptococcus* spp. (i.e. *Streptococcus mitis, Streptococcus sanguinis* and *Streptococcus salivarius*) are able to exclusively bind human alpha-amylase (*AMY1* gene), which is one of the most abundant enzymes in human saliva, through the action of the *abpA* and *abpB* genes [127]. *AMY1* has different copy numbers in modern human populations and is considered to be a genetic trait selected as a result of changes in the dietary choices of our species over time [128]. The ability of human *Streptococcus* spp. to trap human amylase thus suggests how these species have co-evolved in relation to both host genetic and host dietary changes.

### (a) Phylosymbiosis: when host–microbe relationship mirrors host phylogeny

Microbes have shaped the evolutionary landscapes of all multicellular organisms over billions of years. In this context, co-evolutionary patterns have been identified in the case of mutualistic symbioses between animals and their gut microbiome, where their intimate interaction requires mutual adaptations for the benefit of each partner [134]. The overall structure and composition of the gut microbial ecosystem reflect natural selection at both the host and microbial levels, which may result in a functionally stable, co-evolved cooperation characterized by mutual adaptation and benefits [135]. In this scenario, the field of microbiome research has been expanded to include an evolutionary perspective called phylosymbiosis, defined as ‘microbial community relationships that recapitulate the phylogeny of their host’ [136]. When stable, long-term and intimate host–microbe associations occur over evolutionary time, a phylosymbiotic pattern may emerge from co-speciation or co-phylogenetic events, where the two species speciate simultaneously, resulting in parallel evolutionary histories and congruent phylogenies [137]. This may be the case for host–microbe interactions with strong functional interdependence, forcing the two entities to diverge in concert to avoid extinction, and has been described in several insect species that co-evolve and co-speciate with their endosymbiont [138]. One of the first evidences linking phylosymbiotic patterns comes from the cnidarian *Hydra* [139]. In *Hydra*, host-associated bacterial communities reflect the phylogenetic relationship of their host, and knockdown of the *Hydra* arminin family of antimicrobial peptides has been shown to disrupt phylosymbiosis [140]. In *Drosophila*, a recent study has shown that shifts in microbiome composition lead to divergence in *Drosophila* allele frequencies in as few as five generations, with more common alleles in fly populations experimentally enriched for a particular microbial group also being more common in natural populations with a high relative abundance of that microbial group. This suggests that microbiomes could act as a selective force influencing the pattern and process of adaptation even on short timescales [141]. However, patterns of phylosymbiosis are weakly supported in both laboratory strains and wild fruit fly populations, probably owing to *Drosophila*'s constant need to replenish gut microbes from the environment [142]. In social corbiculate bees (honeybees, bumblebees and stingless bees), five core lineages of the gut microbiome show phylogenies that largely match those of the hosts, supporting co-diversification over about 80 Myr [143]. However, some of these bee host lineages have lost or gained gut bacteria over this period, and some members of the bee gut microbiome appear to be opportunistic environmental bacteria or pathogens [144]. Among aquatic invertebrates, several lines of evidence have highlighted phylosymbiotic patterns in sponges, ascidians, crustaceans and corals [145–148]. This contrasts with inconsistent evidence of phylosymbiosis in fishes [74,149].

In great apes, including humans, chimpanzees, gorillas and orangutans, markers from protein-coding genes (sensitive enough to discriminate strains that have diverged over millions of years) provide evidence for co-diversification of hosts and some lineages of gut bacteria, implying long-term vertical association [144,150]. By integrating the existing data describing phylosymbiosis in animal systems, Mallot & Amato found that, while phylosymbiosis appears to become less common as microbiomes become taxonomically richer across the animal kingdom, mammals are the only exception to this general pattern [151]. One possible explanation for this pattern is that in mammals there is a combination of traits that facilitate vertical microbial transmission (i.e. viviparous birth, parental care and milk production) and host control of microbial colonization (adaptive immune system and adaptations to placenta and milk). Although other vertebrates have some of these characteristics, only mammals have all of them [151]. Mammals thus show phylosymbiosis despite having rich symbiotic communities probably because the mentioned host traits offset the stochasticity associated with the dispersal and selection of rich microbiomes. Phylosymbiosis is thus facilitated in mammals because microbial community assembly is less stochastic than in other vertebrates.

Despite these exciting discoveries, recent claims suggest that phylosymbiosis can also arise through simple and non-adaptive host filtering processes. This means that closely related hosts share similar phenotypic traits (e.g. diet, gut pH, gut morphology) that filter similar bacteria present in food and other environmental sources, as seen in *C. elegans* [152] and caterpillars (e.g. *Manduca sexta* species [153]). Within this frame, Groussin *et al*. recently argued that co-evolution is unlikely to explain the co-phylogenetic pattern in the mammalian gut microbiome, and in addition to host filtering effects, the observed co-phylogenetic patterns are likely to result from a geographical model of host speciation with reduced symbiont dispersal and acquisition of symbionts from local species pool [154].

In summary, the extensive research on host–microbe symbioses underscores the significant influence of host genetics on the composition of all animal-associated microbiomes (figure 2), with different taxa showing a consistent correlation between host genotype and gut microbial structure. These findings suggest long-standing and evolving symbiotic relationships between animals and their gut microbes, spanning millions of years, in which hosts act as filters for essential microbes.

## 4. Vertical transmission

Bacterial symbionts can be transferred directly from parent to offspring without mixing with microbes in the environment. This process is called vertical transmission and it plays a crucial role in establishing and shaping the microbial composition and diversity of the gut [155]. It is the primary route by which offspring acquire their first gut microbes. In animals, vertical transmission can occur by a variety of mechanisms, including the transmission through female germ cells (i.e. eggs and embryos), asexual reproduction (e.g. budding in *Hydra*) [156] or direct contact with parents (e.g. via breast milk) [157]. Other indirect mechanisms are also common, including regurgitation of food [158], or egg smearing (and associated behaviours) in insects (box 2). In general, the mode by which a mutualist is transmitted from one generation to the next is strictly related to the importance of the services that it provides to the host [170]. For hosts that show a strong dependence on their symbionts (e.g. endosymbionts), vertical transmission ensures that microorganisms performing critical nutritional functions or other functions essential for their own survival are maintained, while allowing an optimal niche for the symbiont to persist [171]. Many insect species maintain obligate endosymbionts through vertical transmission. This transmission can occur transovarially in the egg, on/near the egg, or by direct association with larvae released into the environment [172]. For example, in carpenter ants, *Blochmania* is transmitted vertically by acute intracellular infection of the ovaries and subsequent incorporation into the eggs [173]. Similarly, in aphids, *Buchnera* is transovarially transmitted to developing eggs through a highly selective mechanism at the ovary tips [174]. In tsetse flies, the B vitamin-supplying symbiont *Wigglesworthia* is transferred via mammary gland secretions during larval development *in utero* [175]. The bacteria *Wolbachia* and *Spiroplasma,* which infect germline tissues intracellularly and/or occur in the haemolymph, have been reported as the only heritable symbionts in *Lepidoptera* and *Drosophila* species [176,177].

Box 2. **Host selection of gut microbes through behavioural features in insects.**

To understand the ecology and diversity of symbionts in insect guts, it is necessary to consider the process of symbiont acquisition. Symbionts can be acquired vertically from mother to offspring with varying degrees of fidelity, horizontally from conspecific or heterospecific individuals, or from the environment. In each of these situations, as we will explain in the following, insects often exhibit sophisticated behaviours to ensure symbiont acquisition. In many hemipteran stink bugs, vertical transmission of gut symbionts requires mothers to smear eggs with symbiont capsules or faecal droplets, from which newborns feed directly [159]. In other hemipterans, however, mothers do not provide such structures, as is the case in the squash bug, *Anasa tristis*. In this species, offspring require bacterial symbionts of the genus *Caballeronia* to ensure successful development, but the bacterium is not provided directly by the females. Instead, offspring are able to locate the symbiont with high fidelity in *A. tristis* faeces or in the environment [160]. Acquisition of gut symbionts from the soil is also common both in caterpillars and stink bugs. Hannula *et al*. [161] showed that the gut microbial communities of caterpillars of the cabbage moth, *Mamestra brassicae*, were similar to those found in soil, but only when caterpillars wandered on the soil surface [161]. This behaviour thus ensures the acquisition of many bacteria, including some defensive ones with known anti-pathogenic properties. Similarly, in the stink bug *Riptortus pedestris,* specific behaviours that allow these insects to acquire bacteria from the soil are key to incorporating bacteria capable of degrading insecticides into their symbiotic repertoire [162,163]. These behaviours include oral acquisition of the bacterium particularly by second- and third-instar nymphs [159]. In herbivores, plants provide an ideal source of symbionts, and phylogenetic evidence has also elucidated the porous nature of symbionts that inhabit both the gut and plant tissues [164,165]. For example, in the beetle *Lagria villosa*, symbionts of the genus *Burkholderia* are required for the development and survival of the insect. These symbionts are related to plant pathogens and they still retain the ability to colonize plants and cause disease. In this species, symbionts are acquired transovarially, but specific behaviours that increase the acquisition of plant-inhabiting strains are likely to allow the incorporation of novel bacterial isolates. Behavioural changes brought by symbionts in social insects can also alter the composition of gut symbionts. For example, symbionts have been reported to reduce aggressive interactions among nest-mates in the leaf-cutting ant *Acromyrmex echinatior* [166] and the German cockroach, *Blattella germanica* [167]. Considering that social contact is one of the main means by which symbionts are acquired in ants, bees, cockroaches and termites [168], reduced aggression could ultimately increase horizontal transmission of symbionts. Altogether, the examples presented here show that many insects acquire mutualistic gut symbionts through sophisticated behaviours, and it is likely that new exciting similar behaviours are yet to be discovered. As the acquired symbionts are in many cases obligate, such a strategy is likely to be efficient in ensuring transmission to the next generation. However, relative to transovarial transmission, this strategy may be less reliable because the wrong symbiont may be acquired, or pathogens may hijack the mode of transmission to ensure persistence. This latter risk has been observed in the trypanosomatid parasite *Leptomonas pyrrhocoris*, which attacks the firebug, *Pyrrhocoris apterus* [169]. However, horizontal symbiont uptake may offer new opportunities, as useful symbionts may be acquired de novo from other species, or from soil or plant surfaces, where they may be pre-adapted to stressors, such as insecticides or extreme abiotic conditions.

However, unlike intracellular symbionts, gut-associated bacteria in insects are generally horizontally acquired during and after birth via horizontal transfer from the surrounding environment. Indeed, *Drosophila* embryos are sterile, but the eggshells carry low microbial loads with high phylogenetic diversity, most likely from adult faeces [142,178], a mechanism also reported in *Heteroptera* [179] and several stink bugs [180]. As larvae consume bacteria from the egg or the environment, the bacterial density in the gut increases throughout the larval stage, reaching a plateau in third-instar wandering larvae [181]. Like *Drosophila*, *C. elegans* harbours a flexible gut microbial pool that is largely dependent on environmental availability. However, recent studies have suggested the presence of a core microbiome in the worm that may be potentially maintained through vertical transmission [182]. Preliminary data also suggest that such transmission may be facilitated by the vitellogenin Vit-2, a family of yolk proteins that are particularly abundant in oviparous animals and are capable of binding bacteria [183]. In most fish, the oviparous behaviour means that gut microbiome symbionts are acquired mainly horizontally from multiple environmental sources, including the eggs, the surrounding water and the first feed [73], although vertical transmission has been reported in some cases [151,184].

By contrast, in humans and other animals (i.e. apes [185], bats [186], mice [187], rats [188], beetles [189]) the consensus on vertical transmission from mother to infant has been largely established by culture-based methods [190], species-level resolution studies [191] and microbial strain-tracking analyses [192]. In humans, mother-to-infant transmission is one of the most influential variables in microbiome composition, as it has been estimated that approximately 50% of the total infant gut microbiome shares exactly the same bacterial strains as the respective mothers [193]. Although the exact timing of the first gut microbial colonizers and the contribution of different sources of microbial seeding have been a matter of debate, it is widely accepted that the first major exposure of the neonate to microorganisms occurs at birth, specifically at the rupture of the amniotic membranes [194]. Numerous studies have also shown that the mode of delivery is a critical factor in determining the early colonization of the neonatal microbiome, with significant differences reported between vaginally and Caesarean-delivered infants. Vaginally delivered infants tend to have microbial communities that resemble those of the mother's vaginal microbiome, whereas Caesarean-delivered infants have bacterial communities (across all body sites) that most closely resemble skin communities [191,195]. Many of these studies have also aimed to assess the functional consequences of differences in vertical microbial transmission, shedding light on various health effects associated with Caesarean section, such as asthma, obesity, allergies and variations in immune system priming [196].

After birth, maternal behaviour and care also strongly promote opportunities for vertical transmission of microorganisms. As all female mammals have mammary glands, milk serves as an additional source of microorganisms that are delivered directly from the maternal body to the infant gut and play a crucial role in directing the early composition, function and metabolism of the infant gut microbiome [191,197]. Furthermore, the direct and prolonged physical contact between mammalian mothers and offspring serves as a means to ensure efficient vertical transmission of oral and gut bacteria [151]. It is important to note that horizontal transmission processes from shared environmental sources to the mother and infant, as well as non-microbial factors present in human milk (e.g. non-digestible carbohydrates, human milk oligosaccharides), also contribute to shaping the infant GI microbiome, specifically supporting the growth of bifidobacterial species [198].

In conclusion, the process of vertical transmission, whereby bacterial symbionts are passed directly from parents to offspring without environmental mixing, is a fundamental mechanism that profoundly influences the establishment and composition of gut microbial communities in a wide range of organisms. This mode of transmission, whether by various mechanisms such as transmission through female gametes, asexual reproduction or direct parent–offspring contact, plays a critical role in ensuring the acquisition of essential gut microbes, particularly in organisms that depend on their symbionts for vital functions (figure 2). While vertical transfer directly influences the passage of essential gut symbionts in some organisms (e.g. bats, apes), others, particularly insects, acquire gut-associated bacteria primarily by horizontal transfer from the environment after birth.

## 5. Immunity

### (a) Innate immunity: the mucosal immune system

The intestinal mucosal immune system is responsible for the interface with the outside world. The microbes of the gut environment constantly shape the immune system, and the host immune system in turn influences the composition of the microbiome. This delicate homeostasis is achieved through an elaborate cross-talk between the microbial activity, the intestinal epithelium and components of the innate and adaptive immune system (e.g. transcriptional regulation, immune effectors) [199]. From an evolutionary perspective, the origin and development of the mucosal surface represents one of the major steps supporting metazoan life and first appeared in members of the Cnidaria [14]. Despite the lack of adaptive immunity, invertebrates have developed sophisticated regulatory mechanisms to tolerate commensal and mutualistic bacteria in the gut while allowing effective immune responses to clear pathogens, which have also been conserved in more complex vertebrates. Within this framework, antimicrobial peptides (AMPs) are evolutionarily ancient molecules that act as key components of innate immunity, and are widely distributed across all species of life, ranging from plants and insects to animals, including molluscs, crustaceans, amphibians, birds, fish and mammals, including humans [200]. Through AMPs, the host controls the community assembly and temporal colonization rate of its microbiome. In the early emerging cnidarian *Hydra*, microbial colonization in early embryos is controlled by maternally encoded AMPs of the periculin family, which are then replaced by zygotically expressed AMPs within three to four weeks as a stable microbiome is established [201] (table 1).

It has been proposed that antimicrobial effectors (i.e. lysozymes, caenopores or saposin-like proteins, caenacins, neuropeptide-like proteins, C-type lectin domain-containing proteins) are also used by the worm *C. elegans* to selectively control its gut microbiota [202,203]. At the same time, members of the nematode's microbial community have been shown to produce protective antimicrobial factors themselves, adding to the nematode's arsenal of effector molecules [9,204].

In *Drosophila*, the key role of AMPs in managing and structuring of the microbiota has been largely demonstrated. Here, AMPs act in concert with other effectors (e.g. lysozyme) to establish and maintain a stable and diverse gut microbiota [205].

AMP expression is regulated by the immune deficiency (IMD)-NF-κB and the Janus kinase–signal transducers and activators of transcription (JAK-STAT) pathways [206]. These signalling pathways are activated by receptors of the peptidoglycan recognition protein (PGRP) family (e.g. PGRP-SD, PGRP-SCs), upon recognition of bacterial peptidoglycan, which can be derived from infecting pathogens as well as commensals [207,208]. IMD mutant flies (Relish-IMD pathway NF-κB mutant flies) have been shown to have higher bacterial loads, altered spatial localization and composition of the microbiota in the gut [209]. Furthermore, in *Drosophila*, additional levels of complexity mediate the regulation of the IMD pathway and immune tolerance to the commensal microbiota. It has been shown that the expression of negative effectors (e.g. pirk, PGRP-LB) downregulates the IMD pathway at virtually all levels of the cascade, and that such regulators are often activated by the IMD pathway itself, establishing a negative feedback loop that adjusts the amplitude of the immune response [208,210]. At the same time, microbiota growth and control is also exerted through compartmentalization of the antimicrobial response, as AMP production is restricted to specific gut segments, through to the mediation of regionalized transcription factors (e.g. caudal) [211]. Strikingly, the intimate evolution of AMP genes (i.e. *diptericin*) with microbiome associates has recently been demonstrated, suggesting that *Drosophila* has adapted its immune repertoire to environmental microbes rather than co-evolving with them [212]. In addition to AMP release, flies also rely on another inducible mechanism, the reactive oxygen species (ROS) production, to control both pathogen invasion and microbiota homeostasis [213]. Specifically, high levels of microbicidal ROS are produced by the dual oxidase DUOX in response to uracil released by pathogens and pathobionts, and flies lacking Duox activity are unable to control mutualistic and pathogenic bacteria, and exhibit a reduced lifespan [26]. Interestingly, the importance of Duox in gut immunity appears to be conserved across different animal model systems, including *C. elegans*, zebrafish, mice and humans [19,34,43].

In vertebrate systems, many other innate immunity-related factors have been shown to play a critical role in maintaining intestinal homeostasis and controlling microbiota composition. Most research efforts to date have focused on pattern recognition receptors (PRRs), which allow the host to recognize microbe-associated molecular patterns (MAMPs) both during inflammation and under steady-state conditions. One of the first discovered PRRs is the Toll-like receptor (TLR), which was first discovered in *Drosophila* but is not directly involved in MAMP recognition in fruit flies [214]. In the cnidarian *Hydra*, conventional TLRs are absent, although a similar LRR domain protein has been shown to interact with a TLR domain-containing protein, the absence of which has been shown to affect microbiota resilience following antibiotic treatment [215]. In *C. elegans*, the only TLR-encoding gene, *tol-1*, is expressed in neurons and is involved in pathogen avoidance behaviour [20]. While the specific mechanisms by which TLRs influence the interaction between invertebrate hosts and their microbiota remain unclear, many studies have characterized their role in mediating non-inflammatory immune responses to the microbiota in mammalian systems, challenging the concept that PRRs have evolved solely to respond to pathogen infection [20]. The first evidence in this sense comes from studies by Rakoff-Nalhoum *et al*. [216], who proposed two distinct TLR functions: host defence against pathogens and control of gut homeostasis through recognition of commensal bacteria [216]. Specifically, TLR2-, TLR4- and Myd88-deficient mice had increased susceptibility to intestinal injury after exposure to dextran sulfate sodium (DSS) as compared with their wild-type counterparts, suggesting that commensal microbiota may be directly recognized by TLRs under homeostatic conditions to mediate a host-protective response [216]. Similarly, TLR5- and TLR1-deficient mice have been shown to exhibit microbiota dysbiosis leading to metabolic syndrome and chronic inflammatory diseases [217].

Among the other PRR molecules that influence gut microbiota, NLRs are of particular importance. In addition to recognizing MAMPs, including bacterial flagellin and peptidoglycan, NLRs also recognize host-derived molecules that are indicative of stress signals (e.g. molecular crystals, potassium efflux, ATP, ROS) [163]. From an evolutionary point of view, the absence of NLRs in invertebrates (e.g. *Drosophila*, *C. elegans*) has led to the assumption that this family of receptors originated in teleost fish [218]. However, subsequent studies suggest that precursors of NLRs already existed in basal metazoans, such as *Hydra magnipapillata*, the sea anemone *Nematostella vectensis*, and the purple sea urchin [219–221], and that the absence of NOD-containing proteins in *Drosophila* and *C. elegans* may indicate that the protostome evolutionary branch lost this family of receptors [222].

In vertebrates, members of the NLR family are highly similar across phyla, with the main differences being in the number of genes [222]. In teleost fish, NOD1 and NOD2 are expressed by the intestinal epithelial cells and promote the expression of DUOX enzymes required for ROS production to limit bacterial numbers [223]. In addition, teleost fish have an additional exclusive group of NLRs, termed NLR group C in zebrafish, which have been proposed to regulate inflammation [224]. Despite this, studies exploring the role of NLRs in teleost fish are limited. In mice and humans, polymorphisms in the *NOD1* and *NOD2* genes have been shown to be associated with dramatic shifts in gut microbiota composition (i.e. increases in Clostridiales, *Bacteroides* spp., segmented filamentous bacteria (SFB) and Enterobacteriaceae), predisposing the host to intestinal inflammation, obesity, diabetes, colorectal cancer and cardiovascular disease [225].

### (b) Adaptive immunity

Despite the importance of elucidating the mechanisms by which microbes are able to colonize their hosts, dissection of the innate and adaptive immune pathways controlling colonization in vertebrates has proven difficult owing to the extensive cross-talk [226]. In this context, it is important to note that the compartmentalization of the immune system into T-cell and B-cell immunity arose at about 500 Ma, probably as a result of the host's need for more specific defence strategies against pathogens [227]. In particular, although invertebrates and vertebrates have similar microbial exposures, there is a greater microbial diversity stably associated with vertebrate hosts than with invertebrate hosts, and it has been suggested that this is a key driver of the evolution of the adaptive immune system in vertebrates, rather than simply a protective mechanism against infection [228]. In this context, cells of the adaptive immune system, in particular CD4+ T cells and mature B cells residing in the gut, have largely been shown to directly control the composition and diversity of symbiotic bacteria. Most research in this area has focused on immunoglobulin A (IgA)-producing B cells, which have been identified as the primary contributors to the maintenance of intestinal homeostasis [199,229]. IgAs are the most abundant immunoglobulin class produced in the mammalian intestinal mucosa. They are relevant factors in the diversity and balance of commensal bacteria by controlling their expansion within the gut, mediating intestinal barrier function, providing a direct protective role against infection and thus maintaining host–commensal interaction [230]. In mice, IgA deficiency leads to an overgrowth of bacterial communities within the gut, with an aberrant expansion of anaerobes and, in particular, spore-forming Gram-positive bacteria (SFB) in the upper segments of the small intestine [231]. Importantly, restoration of normal IgA levels prevents SFB expansion and restores the normal geographical distribution and composition of the gut microbiota [232]. Recent research has sought to understand the mechanisms by which secretory IgA (sIgA) antibodies affect gut bacteria. By generating monoclonal antibodies from plasma cells derived from the intestines of mice colonized with a single bacterial strain (of *Escherichia coli*), the authors showed that approximately 20% of the cloned antibodies reacted with *E. coli* surface antigens and that binding of mouse monoclonal IgAs induced parallel mechanisms, including metabolic changes, protection against bile acids, susceptibility to bacteriophage infection and alteration of motility, that allowed the immune system to exert control in the intestinal lumen and fine-tune host–microbial mutualism [233]. While most of the effects of IgA on the microbiota come from animal studies, the effects of IgA on the human microbiota are not fully understood, although, as in mice, human IgA can respond to gut commensal bacteria [234]. In this context, the finding that cessation of breastfeeding in favour of either formula or food drives the maturation of the infant gut microbiota suggests that the high levels of IgA secreted in breast milk may play a relevant role in regulating the shaping of the gut microbiota [191]. In addition, consistent with the protective role of IgA against pathogens, several human studies have shown that IgA is preferentially bound to pathobionts and/or colitogenic bacteria in inflammatory bowel disease [235]. Although most efforts in the field of host adaptive immunity–microbiota interaction have focused on IgA-secreting B cells, certain subsets of T lymphocytes can also influence microbial composition. Mice lacking a specific subset of T cells (e.g. NKT lymphocytes, lghm^−/−^, Cd3e^−/−^) show microbial dysbiosis, possibly leading to spontaneous colitis, suggesting that T lymphocytes have a B lymphocyte-independent effect on the microbiota in mice [236]. In addition, by transferring bone marrow from Rag1^+/+^ mice to Rag1^−/−^ deficient mice, Zhang *et al*. showed that while Rag1^−/−^ deficient mice were characterized by significantly lower levels of Lactobacillales, complete absence of Enterobacteriales and a consistent increase in *Akkermansia muciniphila* in the colon, adoptive transfer was sufficient to suppress *A. muciniphila* colonization, probably owing to restoration of CD4^+^ T cells and mature B cells [48]. Similarly, transfer of lck : GFP^+^ cells, mainly T lymphocytes, to Rag1-deficient fish resulted in a rapid decrease in intestinal *Vibrio* abundance [237]. This suggests the presence of a population of gut-homing *Vibrio*-specific T cells in these fish that downregulate the numbers of these potentially pathogenic bacteria.

In summary, the complex interplay between the gut immune system and the gut microbiome is a fundamental aspect of host–microbe interactions across species. This dynamic relationship, characterized by bidirectional influences and regulatory mechanisms, underscores the importance of maintaining a delicate balance for overall gut health. In both invertebrates and vertebrates, pattern recognition receptors and antimicrobial effectors play a critical role in shaping and maintaining the composition of the microbiota, contributing to host health and protection against disease. The emergence of the adaptive immune system in vertebrates further highlights the importance of host-specific defence strategies against microbial interactions, ultimately shaping the diversity and stability of the gut microbiota. Understanding these immune–microbiota interactions is crucial for unravelling the mechanisms that drive host health and disease, and offers potential insights for therapeutic interventions and personalized approaches to promote gut homeostasis and overall well-being.

## 6. The importance of environmental factors in shaping the gut microbiota

Beyond host-related factors, gut microbial communities are strongly influenced by ecological dynamics that occur outside an individual host. In this context, community and metacommunity ecology has provided an important theoretical framework by analysing the community-level ecological interactions of host–microbiome systems [238]. Researchers have shown that host-mediated (co-housing, social interactions, physical contact) and microbial interactions predict the taxonomic structure of the gut microbiome in several animal species as well as in humans [239]. Social transmission of microbes can occur through physical social contact (e.g. grooming) and behaviours such as parent–child feeding, mouth-to-mouth interactions between nest-mates, interaction with nest components, and coprophagy (ingestion of faeces) [29,239,240]. In a recent study, Valles-Colomer *et al*. developed strain-tracking analyses to provide a comprehensive view of different patterns of transmission, including vertical transmission and interpersonal horizontal transmission within households and within populations of the gut and oral microbiome. As well as confirming that vertical transmission of gut microbes is stable and long-lasting, the study results also showed that infants lacked many of the bacterial species common in adults, suggesting that these strains are likely to be acquired later via horizontal transmission (i.e. from the environment and social interactions), particularly for the oral microbiome [193].

Host habitat (e.g. geographical location) and priority effects (i.e. the order and timing of past species immigration on the establishment of new species in a community [241]) have recently been shown to be additional important determinants of the gut microbiome in several animal species, including *C. elegans* [242], *Drosophila* [22,243,244], honeybees [245,246], fish [74,77], [247], mice [248] and primates [99,249].

Finally, the host diet is widely recognized as one of the major determinants of the evolution and composition of the gut microbiota in both invertebrates (e.g. gypsy moth larvae [250], cotton bollworm [251], black soldier fly [252], *C. elegans* [253], honeybees [240] and fruit flies [254–256]) and vertebrates (including fish [257], mice [258,259] and humans [121,260]). This is mainly due to the fact that food itself can be a vector for commensals, so that different diets provide microbial inoculants with different community compositions [178]. In this context, Chandler *et al*. found that the microbiota compositions of different *Drosophila* species feeding on the same type of substrate were more similar to each other than to those of more closely related species feeding on different substrates, suggesting that diet overrides host species in shaping microbiota composition [261]. In a seminal study including humans, Ley *et al*. used 16S rRNA analyses of 59 mammalian species and showed that their faecal microbiota clustered according to diet rather than host phylogeny [262]. Similar results were further confirmed by Muegge *et al*., who showed that dietary regimes (i.e. carnivore, herbivore, omnivore) drive convergence across different mammalian lineages, including humans [263].

Taken together, these studies show that most of the microorganisms that colonize the gut of adult animals and humans are largely dependent on environmental factors (e.g. host-to-host contact, dispersal, diet, habitat, etc.). Nevertheless, deterministic processes (e.g. host selection forces) may favour the retention of some microorganisms, meaning that the bacteria obtained from the environment—even the poor colonizers—may evolve to become more prolific colonizers (i.e. host specialists).

## 7. Conclusion

The relationship between animals and microbes is just one of a myriad of evolved symbioses that date back to the dawn of multicellular life [264]. Most studies in this area have focused on how microbes affect the health of animals and humans. However, much less has been done to understand how we affect them. In this review, we summarize our current understanding of the host-related factors that drive the assembly and variation of the gut microbiota in different animals. Our aim is to highlight conserved mechanisms as well as peculiarities and differences specific to some host taxa or species, particularly focusing on model animal species. All animals are under strong natural selection to shape their microbiota to be beneficial, a concept that falls within the holobiont metaphor. Within this framework, we support recent eco-evolutionary theories of the host microbiome as an ecosystem ‘on a leash’ [265]. Specifically, the host can link the fitness of a microbe to the benefits it provides, leading to natural selection for desirable microbial phenotypes. In this review, we have presented the main tools available to the host to target specific strains that commonly act as pathogens to constantly protect itself from invasion while allowing the persistence of commensal or beneficial bacteria. However, the scenario is much more complicated, with neutral forces, environmental fluctuations and constant interactions within the ‘microbial jungle’ playing a major role in shaping the fate of the microbiome. This theory is consistent with a view in which stochastic historical events such as random colonization, random extinction, ecological drift and monopolization, combined with niche pre-emption and modification, drive microbiome variability [248]. The colonizing microbes are then held in place by multiple leashes, from the host and from competing microbes. However, which are the *colonizing* microbes? The most abundant or the most persistent? And what is *persistence* if it can be easily wiped out by pathogenic infection or perturbations?

We believe that much of the research effort should be directed towards understanding how environmental variation shapes microbial response and host control over it, and that in the era of rapid and massive genome sequencing we should not limit research to finding our correlations between host traits and microbial communities. Experimental approaches are important to test hypotheses, which coupled with an assessment of the functional response of microbes and hosts (e.g. spatial transcriptomics and metabolomics) will be powerful tools to understand animal microbiomes at the mechanistic level. Recent advances in molecular editing techniques may also be useful to move the field away from the translocation of gut microbes between species or individuals to the engineering of specific symbiont communities capable of performing pre-designed functions. This will refine our focus on key strains or species and pathways and improve our understanding of host control of microbiome assembly, increasing the possibility of harnessing such control for therapeutic means.

## Data Availability

This article has no additional data.
